# Perceptions of Obesity in Old Age: A Qualitative Study

**DOI:** 10.1192/j.eurpsy.2024.1169

**Published:** 2024-08-27

**Authors:** S. von Humboldt, N. Ilyas, I. Leal

**Affiliations:** ^1^William James Center for Research, ISPA – Instituto Universitário, Lisbon, Portugal; ^2^Center for Clinical Psychology, University of the Punjab, Lahore, Pakistan

## Abstract

**Introduction:**

The relationship between obesity and mental health in old age is complex and widely impacted by different biological, psychological, and social factors.

**Objectives:**

The primary objectives of this qualitative research study are: a) To understand the influence of obesity on older adults’ well-being; b) to assess emotional experiences related to obesity in old age and; c) to explore how obesity influences the mental health of older adults.

**Methods:**

This study included 346 participants aged 65 to 84 years (*M*=73.9; *SD*=5.61) from three different nationalities (English, Spanish, and Portuguese). All interviews went through content analysis.

**Results:**

This study identified four main themes regarding the influence of obesity on older adults’ well-being: (1) Insatisfaction with Body Image (66%); (2) Feeling embarrassed (65%); (3) Feeling Social Isolated (57%); and (4) Lost Opportunities (46%)Three main themes for emotional experiences were frequently verbalized by the participants: (1) Shame (81%); (2) Guilt (78%); and (2) Incompetence (76%). Finally, three main influences in mental health due to obesity were reported: (1) Self-concept (88%); (2) Stress (78%); and (3) Melancholia (63%).

**Conclusions:**

These results highlighted that obesity negatively influences older adults’ well-being and emotional experiences and has serious mental health-related negative outcomes for older adults. Interventions like community-based weight loss programs can be effective in controlling weight and improving the social interaction of obese older adults.

Keywords: Emotional experiences; mental health; obesity; older adults; well-being.

**Disclosure of Interest:**

None Declared

